# Citrus Flavone Tangeretin Inhibits CRPC Cell Proliferation by Regulating Cx26, AKT, and AR Signaling

**DOI:** 10.1155/2022/6422500

**Published:** 2022-01-24

**Authors:** Ningfang Zhang, Wenqi Wu, Yapeng Huang, Lingyue An, Zhichan He, Zhenglin Chang, Zhaohui He, Yongchang Lai

**Affiliations:** ^1^The Eighth Affiliated Hospital of Sun Yat-sen University, Shenzhen, Guangdong 518033, China; ^2^Guangdong Key Laboratory of Urology, Guangzhou 510230, China; ^3^Department of Urology, The Second Affiliated Hospital of Guangzhou Medical University, Guangzhou 510230, China; ^4^Department of Urology, Minimally Invasive Surgery Center, The First Affiliated Hospital of Guangzhou Medical University, Guangzhou Urology Research Institute, Guangzhou 510230, China

## Abstract

Prostate cancer (PCa) progression depends on the action of androgen receptors (AR). Therefore, preventing ligand-mediated activation of AR is the first-line treatment strategy for metastatic PCa. Androgen deprivation therapy (ADT) can inhibit ligand binding to AR and alleviate PCa progression initially. However, due to the adaptation of PCa and recovery of AR signaling, castration-resistant prostate cancer (CRPC) eventually develops. Exploring novel dietary compounds that can target AR signaling appears to be a viable alternative therapeutic option for CRPC. In the present study, compounds from the citrus fruits were focused upon, which contain various flavonoid ingredients. Key components contained within orange peel, which is frequently used in traditional Chinese medicine, and downstream targets were first analyzed using network pharmacology approach. Notably, it was found that tangeretin, an active ingredient from orange peel, can significantly inhibit CRPC cell (C4-2 and Du145 cells) proliferation and migration whilst also synergistically increasing the sensitivity of CRPC cells to anti-tumor drugs sorafenib or cisplatin. Tangeretin also significantly reduced AR and AKT expressions in C4-2 cells and signal transducer and activator of transcription 3 expression in the androgen-insensitive cell line Du145. In addition, tangeretin increased the expression of both connexin26 (Cx26) and gap junction function, which may mediate the bystander effects of cisplatin or sorafenib. Taken together, the present study revealed a novel molecular mechanism by which tangeretin may inhibit the proliferation of CRPC cells, by affecting the Cx26/AKT/AR pathway, to synergistically increase the sensitivity of CRPC cells to sorafenib and cisplatin.

## 1. Introduction

Prostate cancer (PCa) progression depends on androgen receptor (AR) activity [1]. Therefore, preventing the ligand activation of ARs is one of the key treatment strategies for metastatic PCa [1]. Androgen deprivation therapy (ADT) serves to inhibit AR ligand binding and can halt PCa progression initially. However, due to the adaptation of PCa physiology and recovery of AR signaling, a large proportion of patients eventually recur and develop castration-resistant prostate cancer (CRPC) [2]. Regulation of AR signaling during CRPC progression can be mediated by AR signaling amplification and overexpression, AR gene mutations, and AR gene splicing. In particular, splice variants of AR or AR mutants can contribute to resistance to ADT, radiotherapy, or chemotherapy in patients with AR-positive PCa. Although AR inhibitors, such as enzalutamide, abiraterone, and yew alkane drugs, such as docetaxel, are drugs that are commonly available for treating CRPC, therapeutic strategies targeting the full-length AR, AR mutants, and AR-splice variants remained to be unexplored and underdeveloped [3].

Abnormal gap junction (GJ) function has been previously associated with the occurrence, growth, invasion, and metastasis of tumors in addition to resistance to therapy [4]. GJs are assembled by two connexins, which are then assembled into hexamers to form a hemichannel. It allows the transmission of small molecules (<1 kDa), such as ions, metabolites, and even anti-cancer drugs [5]. GJ-mediated exchange of materials between two neighboring cells is termed “gap junctional intercellular communication” (GJIC), which may facilitate the bystander effects of anti-cancer drugs, such as cisplatin [6], sunitinib [7], and dioscin [8]. In addition, connexins can also regulate the occurrence and development of tumors independent of GJ formation [9]. Therefore, GJ/Cx is a potential therapeutic target against cancer metastasis and chemoresistance.

A variety of flavonoids have been reported to exert antitumor effects and can regulate the function of GJ/Cx [10]. Among these, tangeretin has been documented to significantly reverse the impairments in GJIC function induced by tumor promoters such as 12-O-tetradecanoyl-phorbol-acetate (TPA) and 3,5,di-tert-butyl-4-hydroxytoluene (BHT) [11]. Tangeretin is a polymethoxy flavonoid contained within citrus fruit peels and has a molecular weight of 372.37 g/mol, which partially meets the criteria of potential candidate drugs (i.e., the molecular weight is <500 g/mol, the number of hydrogen bond donors is <5, and the number of hydrogen bond receptors is <10). A number of studies have previously shown that tangeretin exerts cytotoxic effects on various cancer cell types [12] and inhibits angiogenesis and metastasis. Furthermore, tangeretin has been reported to scavenge reactive radicals [13] and alleviate inflammation [14]. Cotreatment with tangeretin was also found to not only enhance the sensitivity of hepatoma carcinoma cells, colon carcinoma cells, and ovarian cancer cells to cisplatin [15] but also alleviate kidney damage caused by cisplatin [16] and the carcinogen dimethylbutyric acid [17].

The peel of the orange is rich in polymethoxy flavonoids, whereas citrus is the dry and ripe peel of the orange fruit. Tangeretin has been found to mediate functions of sterilization [18], anti-oxidation, and tumor inhibition [19]. The citrus flavonoid compound hesperetin has been demonstrated to suppress taxane cytotoxicity in prostate cancer cells [20]. In addition, the pectin content of citrus can enhance the sensitivity of prostate cancer to radiation therapy [21]. A previous study investigated the daily fruit and vegetable intake of 142,239 men from 8 countries for 13.9 years, including 7,036 men with prostate cancer [22]. This study found that long-term citrus consumption was associated with a lower risk of prostate cancer [22]. However, for the reason that citrus and orange are rich in a variety of active compounds, the relationship between the different components in citrus and prostate cancer remains unclear. The citrus flavone tangeretin has been found to exhibit biological activities in PC-3 and LNCaP cells by targeting the phosphoinositide 3-kinase (PI3K)/protein kinase B (AKT)/mammalian target of rapamycin (mTOR) and p21 signaling pathways [23, 24]. However, whether the active compounds contained within citrus can inhibit the proliferation of CRPC cells by regulating GJ/Cx, and AR expression remain poorly understood.

In the present study, the network pharmacology analysis was used to screen for the active components and targets within orange peel, following which a series of experiments were performed to verify the results *in vitro*. It was found that tangeretin can inhibit CRPC cell proliferation and increase the potency of cisplatin or sorafenib by modulating the expression of proteins in the Cx26/AKT/AR signaling pathway whilst upregulating the function of GJs.

## 2. Materials and Methods

### 2.1. Pharmacology Analysis and Molecular Docking

Active compounds contained within orange peel were identified using the Traditional Chinese Medicine Systems Pharmacology (TCMSP) database, SymMap database (http://www.symmap.org/), and reported literature. Following absorption, distribution, metabolism, excretion, and toxicity (ADME/T) calculations (oral bioavailability > 20%, druglikeness ＞ 0.1, and permeability Caco-2 ≥ −0.4), compounds were identified to be active and potentially effective compounds. Genes that were predicted to be targeted by orange peel compounds were obtained using the TCMSP, SymMap, and Swiss Target Prediction databases, whereas target genes associated with PCa physiology were obtained using the Online Mendelian Inheritance in Man (OMIM; omim.org/) and GeneCards databases (http://www.genecards.org/). Predicted target proteins were also obtained and converted to genes using the Uniprot database (http://www.uniprot.org/). A citrus-compound-target network was generated using the Cytoscape tool (3.6.0; https://cytoscape.org/). The protein-protein interaction (PPI) network between the potential targets was analyzed using a search tool for retrieval of interacting genes (STRING; http://www.string-db.org) with the species limited to “*Homo sapiens*.” Each node represents proteins produced by a single protein-coding gene locus. Gene ontology (GO) classifications and Kyoto Encyclopedia of Genes and Genomes (KEGG) pathways annotations were searched using the web-based Database for Annotation, Visualization, and Integrated Discovery (DAVID) tool (david.ncifcrf.gov/tools.jsp). The process of molecular ducking was done according to the method described by Yu et al. [25].

### 2.2. Materials

Tangeretin, sorafenib, and cisplatin were purchased from Selleck Chemicals (Houston, USA). Anti-AR, antiphosphorylated (p) AKT, anti-AKT, and antisignal transducer and activator of transcription 3 (STAT3) antibodies were acquired from Cell Signaling Technology (Danvers, USA). The antiactin antibody (GB11001) was purchased from Wuhan Servicebio Technology Co. Ltd. (Wuhan, Hubei, China). The anti-Cx26 antibody (bs-1715R) was obtained from BIOSS (Beijing, China).

### 2.3. Cell Lines and Cell Cultures

CRPC cell lines C4-2 and Du145 cells were obtained from the American Type Culture Collection (Manassas, USA). A-375 cells were purchased from the National Collection of Authenticated Cell Cultures (Shanghai, China). Du145 and A-375 cells were cultured in Dulbecco's Modified Eagle Medium supplemented with 10% fetal bovine serum (FBS, Thermo Fisher Scientific, Waltham, USA), whilst C4-2 cells were cultured in RPMI-1640 supplemented with 10% FBS. All cells were cultured at 37°C and 5% CO_2_ in a humidified atmosphere. To construct Cx26-overexpressing cell lines, a full-length Cx26 cDNA was inserted into the pCMV-MCS-3Flag vector (Beijing Ruiboxingke Biotechnology Co. Ltd., Beijing, China). The transfection procedure was performed in accordance with the instructions of the Lipofectamine® 3000 transfection reagent (Thermo Fisher Scientific, Waltham, USA).

### 2.4. Cell Viability Test

The effect of tangeretin on the proliferation of CRPC cell lines C4-2 and Du145, either alone or in combination with sorafenib or cisplatin, was detected using the 3-(4,5-dimethylthiazol-2-yl)-5-(3-carboxymethoxyphenyl)-2-(4-sulfonphenyl)-2H-tetrazolium (MTS) assay (Beibokit, China). Briefly, after the cells were incubated with the corresponding drugs for 24 h in the corresponding medium, the medium was replaced with a fresh medium (90 *μ*l) containing MTS (10 *μ*l) and incubated in 37°C for 1–3 h. Subsequently, optical values in each well were measured using a multimode reader (BioTek Instruments, Inc., Winooski, USA) at 490 nm. The control group was normalized to “100,” before cell viability was detected and the relative survival rates were evaluated.

### 2.5. Cell Scratch Test

PCa cell migration ability was detected by cell scratch test. After the formation of the cell monolayer, a sterile 200 *μ*l yellow pipette tip was used to scratch the monolayer cells to make a straight line wound. Before the cells were incubated with a fresh cell culture medium containing 1–3% FBS (in a reduced serum condition) and tangeretin, the cells were washed with PBS three times. The nicks were then photographed using microscopy (light).

### 2.6. Colony Formation Assay

Cells suspension with a density of 500 cells/ml was seeded into the culture dish. In total, 24 h later, the medium was replaced with fresh medium containing tangeretin and sorafenib. Subsequently, 10–15 days later, after the macroscopic clones were formed in the culture dish, cells were washed twice with PBS before 4% alcohol crystal violet was added to fix and stain the cells for 20 min. The dye solution was washed away using distilled water, and the cells were air-dried. The colonies were imaged, and then the number of colonies was calculated.

### 2.7. Western Blotting

The protocol of the western blotting was performed according to that described in a previous study [26]. Briefly, after the cells were incubated with the tangeretin and sorafenib for 24 h, the total protein of cells was lysed using RIPA buffer (Thermo Fisher Scientific, Waltham, USA). After the samples were quantified and configured to the same concentration, each lane was loaded with 20 *μ*g total protein, and they were separated by 10%SDS-PAGE; the protein samples were transferred onto polyvinylidene fluoride (PVDF) membranes and blocked by 5% skimmed milk. Anti-Stat3 (1:1,000), pAKT (1:1,000), AKT (1:1,000), Cx26 (1:1,000), and AR (1:1,000) primary antibodies together with their corresponding secondary antibodies (1:2,000, CellSignaling Technology#7074, USA and 1:2,000, CellSignaling Technology#7076, USA) were used to incubate the PVDF membranes. After the PVDF membranes were scanned using the two-color infrared fluorescence protein analysis system (Li-cor Image Studio Ver5.2 software; LI-COR Biosciences, Lincoln, USA), the scanned image was exported before the grayscales were analyzed using the Image J software (National Institutes of Health).

### 2.8. Parachute Dye-Coupling Assay

This assay was used to evaluate the gap junction intracellular communication (GJIC) function as described previously. Donor and receiver cells were first grown to confluence [27]. “Donor cells” were then labeled with 0.5 *μ*mol/l Calcein-AM (Thermo Fisher Scientific, Waltham, USA), which is a membrane dye that can spread to coupled cells. The donor cells were then trypsinized and seeded onto the receiver cells at 1:200 ratios. The donor cells were allowed to attach to the receiver cell monolayer and form GJICs at 37°C for 4 h, before being examined under a fluorescence microscope (Axio Imager A2; Carl Zeiss AG, Oberkochen, Germany). The receiver cells, which received Calcein from donor cells and should emit green fluorescence, were counted visually, and the images were captured. The average number of receiver cells containing Calcein per donor cell was then measured and calculated to deduce the function of GJIC.

### 2.9. Immunofluorescence Analysis

For immunofluorescence imaging, C4-2 cells were cultured in confocal Petri dishes with tangeretin (0, 3, 10, and 30 *μ*m) for 24 h. After three times of PBS rinsing, cells were fixed with 4% paraformaldehyde for 30 min. Before the cells were blocked using 2% bovine serum albumin (BSA) for 30 min under room temperature, the cells were incubated with 0.1% Triton X-100 for 20 min. Subsequently, Cx26 primary antibodies (1:200; Bioss#bs-1715R, China) were applied and incubated overnight in 4°C. After rinsing with PBS, the cells were incubated with Alexa Fluor® 647-conjugated secondary antibodies (1:200; Abcam#ab150075, Cambridge, UK) for 1 h under room temperature in a dark hood. Phalloidin (5 *μ*g/ml) and DAPI (1.43 *μ*M; Abcam#ab1176753, Cambridge, UK) were applied sequentially for actin and nuclear staining, respectively. After another round of PBS rinsing, fluorescent images of the cells were captured under a confocal microscope (LSM880; Carl Zeiss AG, Oberkochen, Germany).

### 2.10. Statistical Analysis

All experiments were repeated at least three times. SPSS 16.0 software (SPSS, Inc., Chicago, USA) was used for the statistical analysis of experimental data. An unpaired *t*-test was used to compare two groups. For two or more groups, one-way ANOVA was used to analyze the data. *P* < 0.05 was considered to indicate a significant difference, where “^∗^” represents a significant difference compared with the corresponding group. The histogram in the graph is expressed in the form of the mean ± standard error.

## 3. Results

### 3.1. Network Pharmacology Study Analysis on the Potential Effects of Orange Peel on PCa

**Figure 1:**
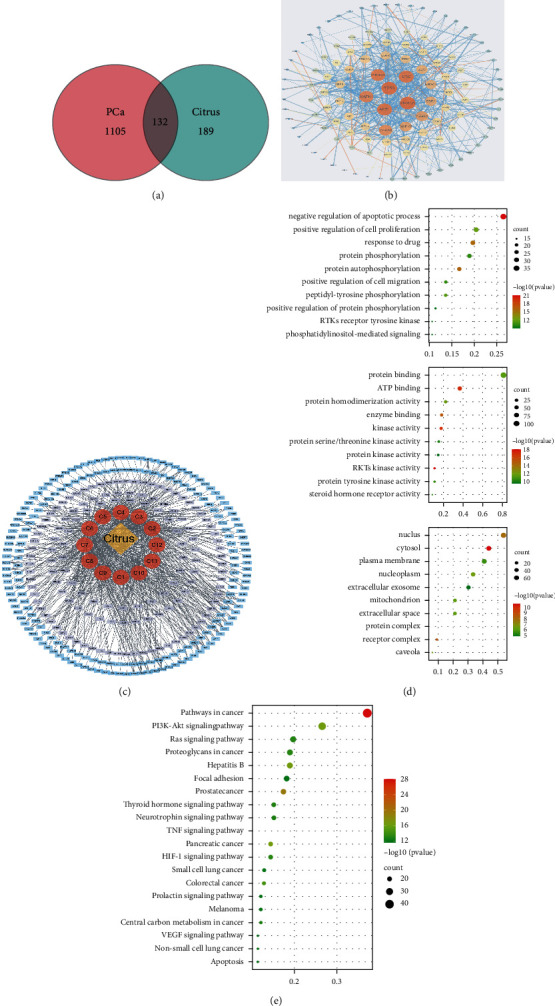
Network pharmacology analysis of the ingredients of orange peel and correlated targets of PCa. (a) Venn diagram showing the visualization of overlapped genes between those targeted by orange peel and those associated with PCa. (b) Protein-protein interaction network of orange peel and their associated targets in PCa. Higher degree values are represented by larger node sizes and brighter colors, whereas higher combined scores are represented by thicker edges and darker colors. Confidence score >0.9. (c) Orange peel-compound-target network. Yellow rhombi, red circles, blue rectangles, and purple rectangles represent orange peel; 12 active compounds were found within the orange peel; and the target genes were targeted by orange peel and overlapped genetic targets between PCa and orange peel, respectively. (d) Scatter plot of the top 10 gene ontology terms in the following three main categories: biological process, cellular component, and molecular function. (e) Scatter plot of the top 20 Kyoto Encyclopedia of Genes and Genomes pathways. The color and size of the dots represent the range of the *Q* values, and the number of differentially expressed genes were mapped to the indicated pathways. PCa, prostate cancer.

Network pharmacology approaches are increasingly being optimized and subsequently applied for exploring novel therapeutic strategies and repurposing previously approved drugs. The present study used this network pharmacology approach to analyze the mechanism underlying the effects of orange peel (citrus) for the potential treatment of PCa. A total of 63 compounds were identified to be associated with citrus from the TCMSP and SymMap databases in addition to the reported articles [28]. Following absorption, distribution, metabolism, excretion, and toxicity (ADME/T) calculations, 12 compounds were identified to be active and potentially effective compounds (Table 1). PCa-associated genes were then obtained from the OMIM and Genecards databases. The Venn diagram revealed the comparison and visualization of genes targeted by orange peel and genes associated with PCa (Figure 1(a)). There are 132 common targets of orange peel and PCa, which are predicted to be key nodes through which citrus exerts its pharmacological effects. To explore these proteins, a PPI network between the orange peel and PCa was constructed and analyzed using STRING with a confidence score of >0.9 (Figure 1(b)). Based on these findings, a citrus-compound-target network was validated (Figure 1(c)). GO analysis was then performed using the web-based DAVID tool, where 561 GO terms were assigned, which included 409 biological processes, 51 cellular components, and 101 molecular function terms (*P* < 0.05). Typically, enriched GO terms are shown in Figure 1(d). The molecular function categories were found to be particularly enriched in “protein binding” (107 differentially expressed genes (DEGs); 81.1%). The highest percentages of GO terms under the cellular component and biological processes were “nucleus” (71 DEGs; 53.8%) and “negative regulation of apoptotic process” (35 DEGs; 16.5%). To identify the signaling pathways involved, KEGG databases were mapped, which found 96 KEGG pathways (*Q* value < 0.01) to be significantly enriched (Figure 1(e)). In particular, the DEGs were found to be highly clustered in a number of signaling pathways, including “pathways in cancer,” “PI3K-AKT signaling pathway,” and “Ras signaling pathways.”

### 3.2. Tangeretin Can Significantly Reduce Cell Viability and Inhibit Colony Formation of CRPC Cells

**Figure 2:**
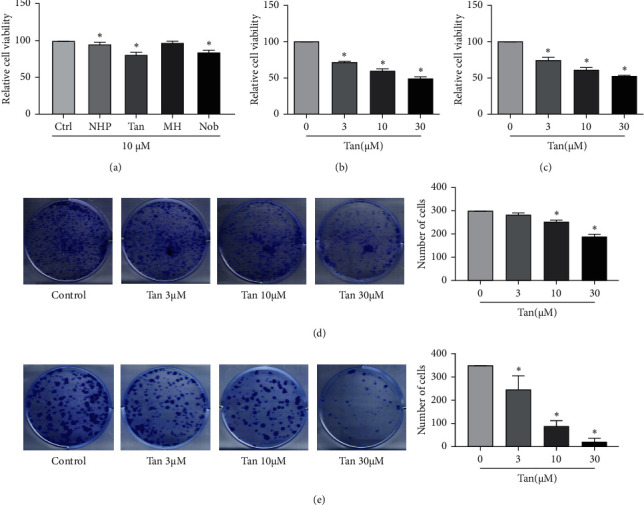
Tangeretin could significantly inhibit CRPC cell viability and colony formation. (a) Following the screening of four active compounds contained within orange peel by using them to treat CRPC cells at 10 *μ*M in 10% FBS, tangeretin was found to be the most effective in reducing C4-2 cell viability. (b) The effect of tangeretin on the viability of Du145 cells in serum-free conditions was detected using an MTS assay. (c) Tangeretin can significantly reduce the viability of C4-2 cells in serum-free conditions. Tangeretin can significantly inhibit colony formation by (d) A-375 and (e) Du145 cells. ^*∗*^*P* < 0.05 compared with the control or the 0 group. CRPC, castration-resistant prostate cancer; Tan, tangeretin; NHP, neohesperidin; MH, methyl-hesperidin; and Nob, 5-Nobiletin.

In total, four compounds were chosen from the list of active components found in the orange peel to treat CRPC cells at the same concentrations (10 *μ*M). Among them, tangeretin was found to be the most effective in inhibiting the cell viability of C4-2 cells in the presence of 10% FBS (Figure 2(a)). Therefore, the effects of tangeretin on CRPC cells were focused upon. The effects of tangeretin on the viability of C4-2 and Du145 cells in serum-free conditions were then assessed using MTS assays. The results revealed that tangeretin can significantly inhibit the viability of Du145 (Figure 2(b)) and C4-2 cells (Figure 2(c)). In addition, tangeretin could significantly inhibit colony formation by A-375 (Figure 2(d)) and Du145 cells (Figure 2(e)).

### 3.3. Tangeretin Can Synergistically Inhibit the Viability of CRPC Cells alongside Cisplatin or Sorafenib *in Vitro*

**Figure 3:**
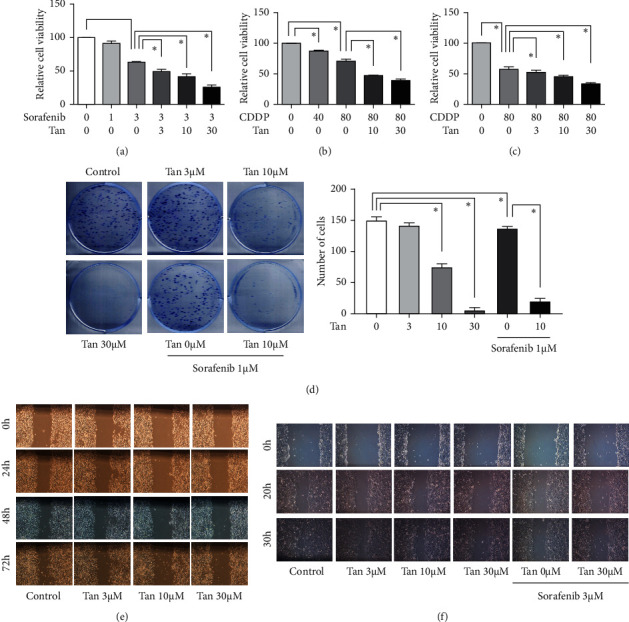
Tangeretin could synergistically inhibit the proliferation and migration of castration-resistant prostate cancer cells when combined with cisplatin or sorafenib under serum deprivation conditions. (a) Tangeretin can synergistically reduce the viability of C4-2 cells when treated alongside sorafenib in serum-free conditions. (b) Tangeretin can also synergistically reduce the viability of C4-2 cells when combined with cisplatin under serum deprivation conditions. (c) Tangeretin can synergistically reduce the cell viability of Du145 cells when combined with cisplatin in serum-free conditions. (d) Tangeretin can significantly inhibit colony formation by C4-2 cells synergy when combined with sorafenib. (e) Tangeretin can significantly inhibit the migration of C4-2 cells (×200). (f) Tangeretin can significantly inhibit the migration of Du145 cells, whilst also synergistically inhibiting their migration when combined with sorafenib under reduced serum conditions (×200). ^*∗*^*P* < 0.05 compared with the control or the 0 group. Tan, tangeretin and CDDP, cisplatin.

Sorafenib and cisplatin are drugs that are typically applied for treating certain types of malignancies [29, 30]. Some drugs can mediate the bystander effect following treatment with cisplatin or other anti-tumor drugs [31–33]. By the way, angiogenesis has a very important role in prostate tumors, and there are several studies showed the correlation [34, 35]. Sorafenib, a tyrosine kinase inhibitor, may exert its antiangiogenic effect [36]. Therefore, the combined effects of tangeretin and sorafenib or cisplatin in CRPC cells were next tested. Tangeretin was found to be able to synergistically inhibit the viability of C4-2 cells (Figure 3(a)) when combined with sorafenib in serum-free conditions. Tangeretin could also synergistically inhibit the viability of C4-2 cells (Figure 3(b)) and Du145 cells (Figure 3(c)) when combined with cisplatin under serum deprivation conditions. In addition, tangeretin synergistically inhibited colony formation by C4-2 cells (Figure 3(d)) when treated alongside sorafenib. As a result, it could be concluded that tangeretin can enhance the inhibitory effects of sorafenib and cisplatin on CRPC cell proliferation.

### 3.4. Tangeretin Can Synergistically Inhibit the Migration of CRPC Cells with Sorafenib *In Vitro*

Wound-healing assays were subsequently performed to explore the effects of tangeretin on the migration of CRPC cells. The results showed that tangeretin can significantly inhibit the migration of C4-2 and Du145 cells (Figures 3(e) and 3(f)) whilst also synergistically enhancing the inhibitory effects of sorafenib on Du145 cell migration.

### 3.5. Tangeretin Inhibits AR, Stat3, AKT, and pAKT Expression in C4-2 Cells and Stat3 and AKT Expression in Du145 Cells

**Figure 4:**
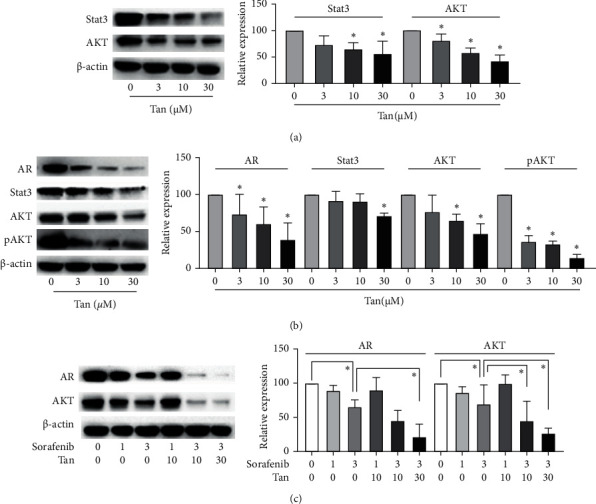
Tangeretin could inhibit AR, Stat3, and AKT expression in castration-resistant prostate cancer cells. (a) Tangeretin inhibits Stat3 and AKT expression in Du145 cells. (b) Tangeretin reduces AR, Stat3, AKT, and pAKT protein levels in C4-2 cells. (c) Tangeretin enhances the inhibitory effects of sorafenib on AR expression in C4-2 cells. ^*∗*^*P* < 0.05 compared with the 0 group. Tan, trangeretin; AR, androgen receptor; Stat3, signal transducer and activator of transcription 3; AKT, protein kinase B; and *p*, phosphorylated.

A series of experiments were then performed to verify the potential effects of tangeretin on the predicted targets according to network pharmacology analysis. It was found that tangeretin could reduce Stat3 and AKT expression in Du145 cells (Figure 4(a)) in addition to inhibiting AR, Stat3, AKT, and pAKT (Ser473) levels in C4-2 cells (Figure 4(b)). As a tyrosine kinase inhibitor, sorafenib can inhibit the PI3K/AKT signaling pathway [37, 38]. Considering that AR plays a key role in prostate cancer, the possibility that tangeretin can synergize with sorafenib to reduce the expression of AR was assessed. It was revealed that tangeretin could synergize with sorafenib to inhibit AR and AKT expression in AR-positive C4-2 cells (Figure 4(c)).

### 3.6. Molecular Docking Analysis of Tangeretin AR and Cx26

**Figure 5:**
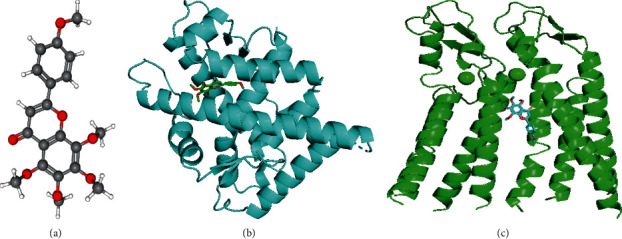
Molecular docking analysis of tangeretin, AR, and Cx26. (a) Molecular structure of tangeretin. (b) Molecular docking of tangeretin onto the crystal structure of AR complexes (PDB ID: 5VO4) with a docking score of –3.0. (c) Molecular docking of tangeretin onto the crystal structure of Cx26 complexes (PDB ID: 5ER7) with a docking score of –6.3. The dotted green line represents hydrogen bonding. AR, androgen receptor and Cx26, connexin 26.

To examine the molecular characteristics of tangeretin, the structures were obtained from PubChem (https://pubchem.ncbi.nlm.nih.gov/;Figure 5(a)). The crystal structures of AR (PDB ID: 5VO4) and Cx26 (PDB ID: 5ER7) were obtained from the PDB database (https://www.rcsb.org/); both structures were complexed with small molecules. The Autodock Vina was used to verify the aforementioned targets after uploading the three-dimensional structure of tangeretin. Through molecular docking, the interaction between tangeretin and the crystal structure of AR complexes (Figure 5(b)) was verified, yielding a docking score of –3.0. In addition, interaction between tangeretin and the crystal structure of Cx26 complexes (PDB ID: 5ER7), a gap junction protein that can mediate the “bystander effect” by transmitting “death signals” to adjacent cells to enhance the killing effect of chemotherapy [39], was verified, which yielded a docking score of –6.3 (Figure 5(c)). These results suggest that tangeretin can directly interact with AR and Cx26 through hydrogen bonding.

### 3.7. Tangeretin Increases Cx26 Expression and GJIC

**Figure 6:**
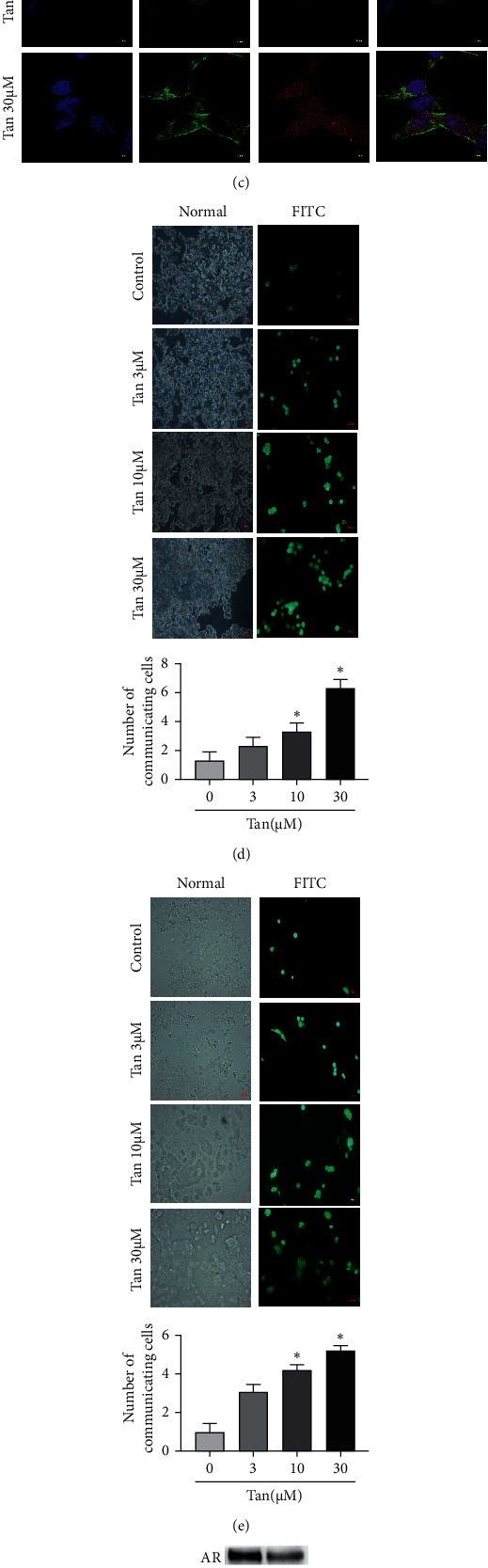
Tangeretin increased Cx26 expression and facilitated AKT and AR signaling to promote GJIC function in castration-resistant prostate cancer cells. (a) Tangeretin can significantly increase Cx26 expression in C4-2 cells. (b) Tangeretin can significantly increase Cx26 expression in Du145 cells. (c) Immunofluorescence analysis confirmed that tangeretin can increase Cx26 expression (10 × 100). (d) Functional GJIC was detected using parachute assay in C4-2 cells (×200). (e) Functional GJIC was detected using parachute assay in Du145 cells (×200). (f) Cx26 overexpression can significantly inhibit the expression of AKT and AR in C4-2 cells. (g) Cx26 overexpression can reduce the viability of C4-2 cells as detected by MTS assay 72 h after transfection. (h) Representative western blotting images and quantification of Cx26, AKT, and AR protein expression in C4-2 cells and Cx26-overexpressing C4-2 cells. (i) Protein-protein interaction network of tangeretin and target genes following search tool for the retrieval of interacting genes/proteins analysis. Confidence score >0.4. ^*∗*^*P* < 0.05 compared with the control or the 0 group. Tan, tangeretin; Cx26, connexin 26; AKT, protein kinase B; AR, androgen receptor; and GJIC, gap junctional intercellular communication.

To verify the aforementioned molecular docking results, Cx26 expression was measured by western blotting, where the results revealed that tangeretin significantly increased Cx26 expression in both C4-2 and Du145 cells (Figures 6(a) and 6(b)). In addition, Cx26 expression and distribution were evaluated by immunofluorescence staining. The result showed that tangeretin treatment significantly increased the expression of Cx26 in C4-2 cells compared with that in untreated control cells (Figure 6(c)). The reduction or disappearance of GJs has been frequently associated with the development and growth of tumors [4]. Restoring GJ function in tumor cells can inhibit the proliferation of tumor cells by reprogramming the regulatory mechanism [40]. Therefore, GJIC function was next detected using parachute assay, which found that tangeretin treatment enhanced GJIC function among C4-2 cells (Figure 6(d)) and Du145 cells (Figure 6(e)). Taken together, tangeretin could significantly increase Cx26 expression whilst enhancing GJIC in CRPC cells.

### 3.8. Cx26 Overexpression Inhibits AKT and AR Expression

To decipher the associations and interactions among the aforementioned molecules, Cx26-overexpressing cell lines of C4-2 were constructed. The results showed Cx26 overexpression could inhibit the expression of AKT and AR (Figures 6(f) and 6(i)) whilst also reducing the viability of C4-2 cells (Figure 6(g)).

According to the target genes of tangeretin, a PPI network was constructed using the STRING tool (Figure 6(h)). From the PPI network, although the predicted direct relationships among the molecules aforementioned could be observed, Cx26 was not found to be one of the core molecules according to these predictions based on previous data. However, this observation provided a novel perspective on the role of Cx26 in AKT and AR expression.

Taken together, data from the present study revealed that tangeretin can inhibit the proliferation of CRPC cells by upregulating Cx26 expression, which in turn inhibited AKT and AR expression to synergistically enhance the sensitivity of CRPC cells to cisplatin and sorafenib.

## 4. Discussion

In the present study, the key components and targets of orange peel, a traditional Chinese medicine, were first analyzed using a network pharmacology method. This screened out 12 predicted compounds contained within orange peel from the TCMSP database, where their corresponding targets were subsequently predicted. By integrating these with the PCa-associated disease genes, 132 overlapped genes and drug targets were found. To confirm the relationship of these compounds further with PCa, a corresponding compound-target-signaling pathway network was also constructed. It was found that tangeretin, an active ingredient from orange peel, could significantly inhibit CRPC cell (C4-2 and Du145) proliferation and migration, whilst synergistically enhancing their sensitivity to sorafenib and cisplatin. Tangeretin also significantly reduced AR and AKT expression in C4-2 cells in addition to reducing Stat3 in androgen-insensitive Du145 cells.

Additionally, tangeretin increased the expression of both Cx26 and enhanced GJ function in CRPC cells, which may mediate the bystander effect following treatment with cisplatin or other anti-tumor drugs [31–33]. Therefore, Cx26-overexpressing cell lines were then constructed, where it was found that Cx26 overexpression inhibited AR and AKT signaling. Therefore, Cx26 may serve to be a novel target that can be manipulated to hinder the development and progression of PCa and AKT/AR signaling (Supplementary Figure 1).

Almost all mammalian cells are connected through GJs. Small molecules, such as calcium ions, IP_3_, and cAMP, can be transmitted among cells through GJs, such that cells can share metabolites to regulate the signal transmission status of each other. GJ serves an important role in the occurrence, growth, invasion, and metastasis of tumors in addition to the response to therapy. During tumor chemotherapy or radiotherapy, cells can transmit “death signals” to adjacent cells by GJs to promote apoptosis, thereby enhancing the killing effect, in a phenomenon known as the “bystander effect” [41, 42]. Through molecular docking analysis, it was revealed that Cx26, a gap junction protein, may be a target of tangeretin. Cx26 genes are located on chromosome 13 [43]. It is mainly expressed in epithelial cells and serves an important role in maintaining the normal flow of materials and signals among cells in the urothelial tract. In particular, there are large quantities of glandular and basal epithelial tissues in the prostate.

Previous studies have shown that the expression levels of Cx26 were negatively correlated with the malignancy severity of transitional cell carcinoma [44]. Transfection with the Cx26 adenovirus was found to inhibit the proliferation of a variety of PCa cell lines *in vitro*, such as PC-3, LNCAP, and DU-145, by inducing cell cycle arrest at the *G*_2_ or *M* phases to promote apoptosis [45]. In addition, Cx26 can reduce the invasion and metastasis of tumor cells by inhibiting adhesion kinase adhesion [46]. The present study also revealed that tangeretin can inhibit the proliferation of CRPC cells by inducing Cx26-mediated inhibition of AKT and AR signaling whilst facilitating the formation of GJIC. In addition, the GJIC formed by Cx26 could promote the “bystander effect” following treatment with sorafenib or cisplatin. Therefore, both junctional and nonjunctional inhibitory effects mediated by Cx26 were presented. Since Cx26 expression could inhibit the proliferation of CRPC cells, the specific role of Cx26 in the development of PCa is anticipated to be unraveled by future investigations.

By regulating nutrient metabolism, cell proliferation, survival, migration, and angiogenesis, the PI3K/AKT signaling pathway was found to be activated in a wide variety of cancer types [47]. AKT/mTOR [48] and AR signaling pathways [49] are aberrantly activated in prostate cancer, where the mechanism between the PI3K/AKT/mTOR pathway and several key oncogenic signaling cascades, such as AR, mitogen-activated protein kinase (MAPK), and Wnt signaling cascades [50], underlying PCa growth and drug resistance has been previously discussed [51]. Tangeretin has been reported to inhibit the proliferation of PCa cells by targeting the PI3K/AKT/mTOR signaling pathway [24]. The present study therefore selected two CRPC cell lines, where the results not only confirmed AKT inhibition by tangeretin but also discovered that tangeretin can inhibit AR expression, which serves a key role in the development of PCa and CRPC. However, tangeretin has been found to have inhibitory effects on a variety of tumors [12, 52], it remains to be further explored whether tangeretin can affect the progression of other tumors by inhibiting AR expression and whether it can also exert inhibitory effects on other tumors through Cx26/AKT/AR signaling pathway.

AR signaling is a hub that laid the foundation for a number of signaling mechanisms in CRPC, where it serves a central role in mediating the process of androgen-dependent progression to CRPC. The mechanism underlying AR triggering in CRPC mainly includes AR point mutations, AR overexpression, and AR coactivators, all of which promote the abnormal activation of AR [53]. Therefore, AR is also a therapeutic target for CRPC. Apart from the AR signaling pathway, receptor tyrosine kinases and AKT1 have also been previously associated with PCa [54]. AR mutations frequently occur in the ligand-binding domain (LBD) of AR, where the most common mutation is the T877 A mutation [55]. However, the role of tangeretin on AR full length (AR-FL) and AR-splice variants (AR-Vs) require further investigation.

In the present study, tangeretin was selected to be the key research target from the orange peel. Another citrus compound naringenin was found to induce apoptotic cell death in PCa cells via the PI3K/AKT and MAPK signaling pathways [56], which is consistent with the present study. At present, the protective effects of citrus on PCa have been reported by epidemiological analysis, but the corresponding effects of citrus and tangeretin on CRPC in adjuvant diet regimen require further verification by additional clinical evidence. As one of the active ingredients of orange peel that is readily available and more economically viable than abiraterone, tangeretin can be used as a dietary supplement in daily life, which may serve as a synergistic treatment strategy whilst reducing toxic side effects and preventing drug resistance in patients.

However, more experimental and clinical evidence were needed to determine the relationship between tangerine peel and citrus and prostate cancer. In addition, due to the influence of water solubility, the antiprostate cancer activity and bioavailability of citrine still need to be further optimized. The development of AR-targeting drugs based on tangeretin and orange peel may reduce the treatment burden of patients with PCa to some extent. The structural modification and exploitation of using tangeretin to target AR may provide a novel direction for the development of dietary compounds for PCa treatment.

## 5. Conclusion

The present study revealed that orange peel and its active compound tangeretin can inhibit the proliferation, migration, and colony formation of CRPC cells. In addition, strong indications were provided that tangeretin can inhibit AR expression, which serves a key role in the development of PCa. By inducing the expression of Cx26, tangeretin can inhibit AKT and AR expression whilst facilitating the formation of GJIC. These results may have an impact on the therapy of CRPC and for the food and medicine industry with regard to using orange peel.

## Figures and Tables

**Table 1:** The 12 active compounds in citrus.

No.	Compound	OB (%)	DL	CaCo_2_
C1	Ledene	51.84	0.1	1.86
C2	Sitosterol	36.91	0.75	1.32
C3	Naringenin	59.29	0.21	0.28
C4	Hesperetin	47.74	0.27	0.28
C5	DIBP	49.63	0.13	0.85
C6	Hepta-3	23.91	0.58	1.05
C7	Tangeretin	21.38	0.43	1.23
C8	Citromitin	86.9	0.51	0.88
C9	Nobiletin	61.67	0.52	1.05
C10	Hesperidin	13.33	0.67	−2.03
C11	Neohesperidin	11.57	0.69	−2.05
C12	Hesperetin-5-glucoside	21.82	0.83	−1.42

## Data Availability

The data used to support the findings of this study are included within the article.

## Abbreviations

ADPC: Androgen-dependent prostate cancer
Akt: Protein kinase B
AR: Androgen receptor
CRPC: Castration-resistant prostate cancer
Cx26: Connexin 26
GJIC: Gap junction intercellular communication
Tan: Tangeretin.

## Supplementary Materials

Supplementary Materials
*Supplementary Figure 1.* Tangeretin significantly inhibits AR and AKT expression in CRPC cells. Intriguingly, tangeretin increases both the expression of the connexin26 (Cx26) and the function of gap junction, which may mediate the bystander effect of antitumor drugs.
