# Supplementary material for: Citrus Flavone Tangeretin Inhibits CRPC Cell Proliferation by Regulating Cx26, AKT, and AR Signaling
Source: Evid Based Complement Alternat Med. 2022 Jan 24;2022:6422500. doi: 10.1155/2022/6422500 (PMC8803427; doi:10.1155/2022/6422500)
Supplement: Supplementary Materials — Supplementary Figure 1. Tangeretin significantly inhibits AR and AKT expression in CRPC cells. Intriguingly, tangeretin increases both the expression of the connexin26 (Cx26) and the function of gap junction, which may mediate the bystander effect of antitumor drugs. [file 6422500.f1.pptx]

## Slide 1
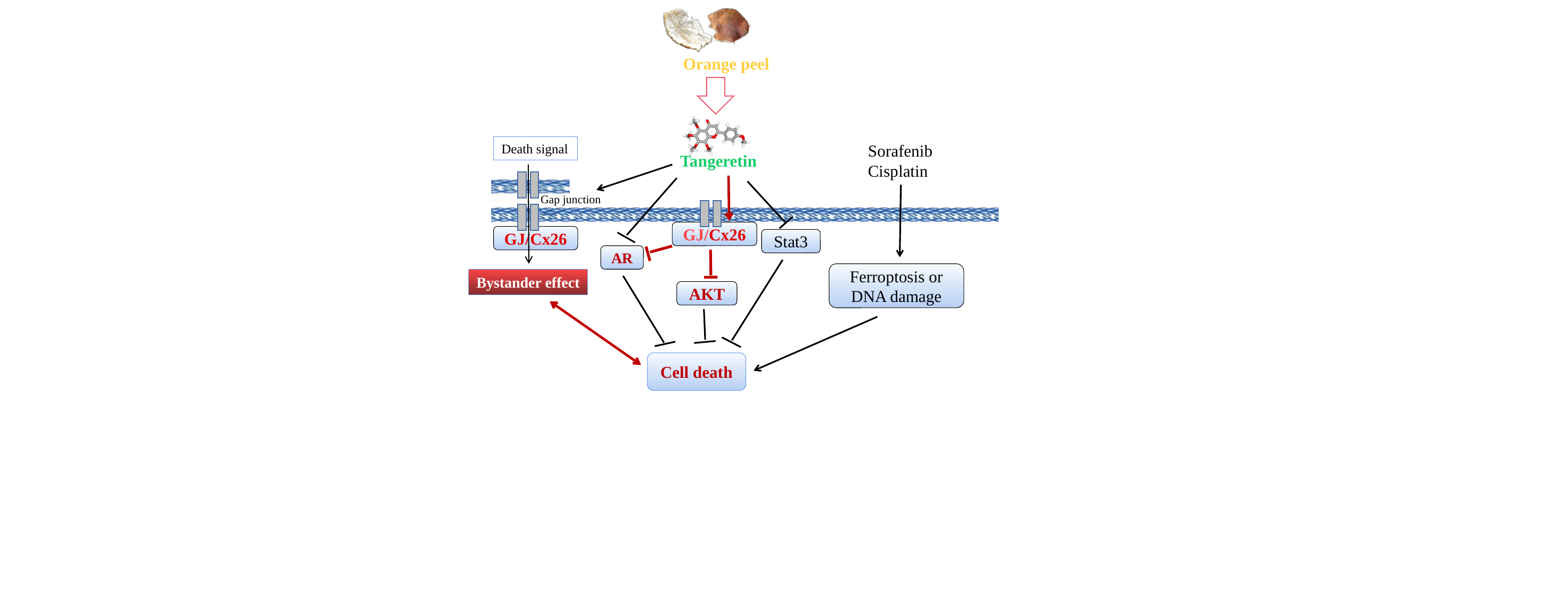

Orange peel
Death signal
SorafenibCisplatin
Tangeretin
Gap junction
GJ/Cx26
GJ/Cx26
Stat3
AR
Ferroptosis or DNA damage
Bystander effect
AKT
Cell death
